# The association of iron status on clinical outcomes in peritoneal dialysis: a retrospective study over 10 years

**DOI:** 10.1080/0886022X.2025.2567522

**Published:** 2025-10-09

**Authors:** Vanessa Wing-Lam Tao, Jack Kit-Chung Ng, Winston Wing-Shing Fung, Gordon Chun-Kau Chan, Kai-Ming Chow, Cheuk-Chun Szeto

**Affiliations:** ^a^ Departments of Medicine and Therapeutics, Prince of Wales Hospital, Carol & Richard Yu Peritoneal Dialysis Research Centre, Hong Kong SAR, China; ^b^ Li Ka Shing Institute of Health Sciences (LiHS), Faculty of Medicine, The Chinese University of Hong Kong, Shatin, Hong Kong SAR, China

**Keywords:** Anemia, inflammation, chronic kidney disease, cardiovascular disease

## Abstract

Iron deficiency and overload are common in kidney disease. Although the relation between iron status and clinical outcome is well reported in hemodialysis, there are few publications in peritoneal dialysis (PD). We investigated the association between iron status and outcomes of new PD patients. We reviewed 1,804 new adult PD patients between 2011 and 2020. Their baseline status was classified into reference iron status (RIS), absolute iron deficiency (AID), low iron storage (LIS), functional iron deficiency (FID), and high iron status (HIS) according to the transferrin saturation and serum ferritin level. After a median follow-up of 35.2 months, outcome measures, including patient survival, technique survival, peritonitis-free survival, number of hospital admission, and length of hospitalization, were analyzed. FID and HIS were found in 18.8% and 57.5% patients, respectively. The 2-year patient survival for RIS, AID, LIS, FID, and HIS groups were 87.4%, 90.2%, 94.7%, 82.0%, and 83.5%, respectively (log-rank test, *p* < 0.001). However, all-cause mortality rate was not significantly different between iron status groups after adjusting for clinical confounders. The FID group was associated with more hospital admission than the other groups, but the difference became insignificant after adjusting for clinical confounders. In conclusion, FID and HIS are common in incident PD patients. FID was associated with a trend of lower patient survival and higher hospitalization rate, but the differenced were not significant with multi-variable analysis. Further studies are required to determine the desirable iron saturation or ferritin level to guide iron replacement therapy.

## KEY LEARNING POINTS

**What was known:**
  The relation between iron status and clinical outcome is well reported in hemodialysis.
**This study adds:**
  Functional iron deficiency (FID) and high iron status (HIS) are both common in peritoneal dialysis (PD) patients.
  All-cause mortality rate was not significantly different between iron status groups after adjusting for clinical confounders.
**Potential impact:**
  Further studies are required to determine the desirable iron saturation or ferritin level to guide iron replacement therapy.

## Introduction

Anemia is commonly found in patients with chronic kidney disease (CKD), and is associated with increased mortality, cardiovascular disease, cognitive impairment and hospitalizations [1]. Intravenous (IV) and oral iron therapy, erythropoietin-stimulating agents (ESA), and hypoxia-inducible factor–prolyl hydroxylase (HIF-PHI) inhibitors have been widely used to tackle anemia in CKD patients. Iron deficiency has been considered as a major cause of inadequate response to treatment with erythropoiesis-stimulating agents (ESAs). In addition to absolute iron deficiency (AID), functional iron deficiency (FID) was characterized by impaired iron release from body stores, with a low serum transferrin saturation (TSAT) and normal or high serum ferritin [2]. It was shown that FID was associated with increased mortality in non-dialysis dependent chronic kidney disease patients [3,4]. However, limited data were available regarding optimal ferritin level and association in clinical outcomes of patients receiving peritoneal dialysis (PD). A study from Guangzhou reported a significant association between FID and all-cause mortality risk among a cohort of PD patients [5]. Another study from Guangzhou showed AID and HIS were associated with more incidence and treatment failure of peritonitis [6].

Although the relation between iron status and the clinical outcome has been well reported in hemodialysis patients [7,8], there are few publications in patients receiving PD. The aim of this study is to understand the prevalence of iron deficiency and overload in incident PD patients, as well as to explore the prognostic significance of baseline iron status on the outcome of PD patients.

## Methods

The study was approved by the Joint Chinese University of Hong Kong – New Territories East Cluster Clinical Research Ethics Committee (approval numbers CREC-2023.363). All study procedures followed the Declaration of Helsinki.

### Study population and data collection

This was a retrospective cohort study. We reviewed 2029 adult patients who were started on PD in our unit from 1 January 2011 to 31 December 2020. Their baseline demographic and clinical data were retrieved from the Clinical Data Analysis and Reporting System (CDARS). The result of iron profile, including serum iron level, total iron binding capacity (TIBC), TSAT, and serum ferritin level, at the initiation of PD was reviewed. Other baseline clinical and laboratory parameters, including Charlson Comorbidity Index (CCI), total weekly Kt/V, residual glomerular filtration rate (GFR), thalassemia trait status, hemoglobin (Hb) level, mean corpuscular volume (MCV), calcium, phosphate, albumin, fasting glucose (FG), HbA1c (which was measured in all patients irrespective to their diabetes status), low-density lipoprotein cholesterol (LDLc), triglycerides (TG), vitamin B12, folate, and C-reactive protein (CRP) were reviewed. The use of IV iron, oral ferrous supplement and ESA at the initiation of PD was recorded. Transfusion at baseline were counted if the patient received transfusion within 90 d before the date of start of dialysis. In general, blood transfusion was given when there was acute blood loss of >20% of the blood volume, Hb level < 7 g/dL, or Hb level 7–10 g/dL when there were anemic symptoms.

### Definition and outcomes

Iron status was classified into different categories as previously described by Luo et al. [5]: reference iron status (RIS) (TSAT ≥ 20% and ferritin 100–500 ng/mL); AID (TSAT < 20% and ferritin < 100 ng/mL); low iron storage (LIS) (TSAT ≥ 20% and ferritin <100 ng/mL); functional iron deficiency (FID) (TSAT < 20% and ferritin > 100 ng/mL); and high iron status (HIS) (TSAT ≥ 20% and ferritin > 500 ng/mL).

All patients were followed till 31 December 2020. Outcome measures of this study include patient survival, technique survival, peritonitis-free survival, number of hospital admission, and length of hospitalizations for all cause. For patient survival, death within 3 months after conversion to hemodialysis or transplantation were counted as events. For the analysis of technique survival, the follow up was censored for transferal to other centers, loss to follow up, or recovery of renal function.

### Statistical analyses

Statistical analysis was performed by SPSS for Windows software version 27.0 (IBM, Armonk, NY). The baseline demographic data and biochemical parameters of different iron status groups were compared by chi-square test, one-way ANOVA, or Kruskal–Wallis test as appropriate. Kaplan–Meier plots were constructed for patient survival, technique survival, and peritonitis-free survival, and iron status groups were compared by log-rank test. Cox proportional hazard models were then constructed to estimate the associations between iron status groups and patient survival, technique survival, and peritonitis-free survival. The results were expressed in hazard ratio (HR) and 95% confidence interval (CI). The number of hospitalizations and length of hospitalization were adjusted for the duration of follow up and compared between iron status groups by the Kruskal–Wallis test. Multiple linear regression models with log transformation of the hospitalization data were then performed to adjust for other clinical confounding factors on the association between baseline iron status and hospitalization. For the Cox regression and linear regression analyses, covariates included age, sex, total weekly Kt/V, residual GFR, CCI, thalassemia trait status, Hb level, MCV, calcium, phosphate, albumin, HbA1c, LDLc, TG, vitamin B12, folate, and CRP levels, recent treatment of IV or oral iron, the use of ESA, and transfusion within 90 d before start of dialysis. A *p* value of < 0.05 was considered statistically significant. All probabilities were two-tailed.

## Results

### Baseline characteristics

We identified 2029 new PD patients in our unit from 2011 to 2020; 225 were excluded because of incomplete data on their iron status. We analyzed the remaining 1,804 patients, and they were classified according to their iron status: 365 (20.2%) were in the RIS group, 43 (2.4%) had AID, 19 (1.1%) had LIS, 339 (18.8%) had FID, and 1,038 (57.5%) had HIS. The baseline demographic data and biochemical parameters are shown in Tables 1 and 2, respectively. In essence, the FID group were more likely to have diabetes, higher CCI, and serum CRP level than the other groups. In contrast, the HIS group had the lowest residual GFR, Hb, and albumin levels than the other groups. More patients with FID received oral iron supplement, while more patents with HIS received transfusion. The use of IV iron and rhEPO was similar between groups. None of the patient received hypoxia-inducible factor prolyl hydroxylase inhibitor (HIF-PHI) therapy.

**Table 1.** Baseline demographic data of individuals stratified by iron status at the start of peritoneal dialysis^a^.

Iron status	All patients	Reference iron status	Absolute iron deficiency	Low iron storage	Functional iron deficiency	High iron status	*p* Value
No. of patients	1804	365	43	19	339	1038	0.254
Age (years)	63.79 ± 11.42	63.58 ± 11.05	60.58 ± 13.21	64.58 ± 13.52	64.60 ± 10.48	63.72 ± 11.72	0.254
Sex: Male (*n*, %)	1121 (62.1%)	217 (59.5%)	14 (32.6%)	10 (52.6%)	211 (62.2%)	669 (64.5%)	**<0.001**
Type: CAPD (*n*, %)	1451 (80.4%)	296 (81.1%)	28 (65.1%)	13 (68.4%)	276 (81.4%)	838 (80.7%)	0.115
Cause of ESKF (*n*, %)							**<0.001**
DM	947 (52.5%)	192 (52.6%)	15 (34.9%)	6 (31.6%)	216 (63.7%)	518 (49.9%)	
GN	231 (12.8%)	73 (20.0%)	15 (34.9%)	8 (42.1%)	29 (8.6%)	223 (21.5%)	
HT	348 (19.3%)	46 (12.6%)	7 (16.3%)	1 (5.3%)	43 (12.7%)	134 (12.9%)	
PKD	44 (2.4%)	15 (4.1%)	1 (2.3%)	2 (10.5%)	5 (1.5%)	21 (2.0%)	
Urological	35 (1.9%)	7 (1.9%)	0 (0.0%)	1 (5.3%)	4 (1.2%)	23 (2.2%)	
Others	75 (4.2%)	13 (3.6%)	4 (9.3%)	1 (5.3%)	18 (5.3%)	39 (3.8%)	
Unknown	124 (6.9%)	19 (5.2%)	1 (2.3%)	0 (0.0%)	24 (7.1%)	80 (7.7%)	
Major comorbidities (*n*, %)							
DM	1092 (60.5%)	215 (58.9%)	21 (48.8%)	10 (52.6%)	253 (74.6%)	593 (57.1%)	**<0.001**
IHD	323 (17.9%)	65 (17.8%)	8 (18.6%)	3 (15.8%)	65 (19.2%)	182 (17.5%)	0.969
CVA	256 (14.2%)	46 (12.6%)	5 (11.6%)	5 (26.3%)	45 (13.3%)	155 (14.9%)	0.408
Thal trait	42 (2.3%)	8 (2.2%)	1 (2.3%)	0 (0.0%)	9 (2.7%)	24 (2.3%)	0.898
CCI	6.16 ± 2.25	6.13 ± 2.20	5.51 ± 2.52	6.26 ± 2.73	6.53 ± 2.12	6.08 ± 2.27	**0.006**
IV iron use* (*n*, %)	87 (4.8%)	9 (2.5%)	3 (7.0%)	0 (0.0%)	19 (5.6%)	56 (5.4%)	0.068
Oral iron use* (*n*, %)	650 (36.0%)	124 (34.0%)	16 (37.2%)	5 (26.3%)	158 (46.6%)	347 (33.4%)	**<0.001**
EPO use* (*n*, %)	977 (54.2%)	205 (56.2%)	29 (67.4%)	8 (42.1%)	196 (57.8%)	539 (51.9%)	0.070
Transfused^#^ (*n*, %)	798 (44.2%)	92 (25.2%)	8 (18.6%)	3 (15.8%)	141 (41.6%)	554 (53.4%)	**<0.001**

CAPD: continuous ambulatory peritoneal dialysis; ESKF: end-stage kidney failure; DM: diabetes mellitus; GN: glomerulonephritis; HT: hypertension; PKD: polycystic kidney disease; CCI: Charlson Comorbidity Index; IHD: ischemic heart disease; CVA: cerebral vascular accident; Thal trait: thalassemia trait; IV iron: intravenous iron; EPO: erythropoietin

*Within 90 d before/after start of dialysis. ^#^6 months around the start of dialysis. ^a^See text for the definition of each iron status group.

The bold values indicates statistical significance.

**Table 2.** Baseline biochemical parameters of individuals stratified by iron status at the start of peritoneal dialysis^a^.

Iron status	All patients	Reference iron status	Absolute iron deficiency	Low iron storage	Functional iron deficiency	High iron status	*p* Value
Kt/V	2.13 ± 0.66	2.25 ± 0.76	2.31 ± 0.60	2.17 ± 0.64	2.15 ± 0.65	2.09 ± 0.64	0.077
rGFR, mL/min/1.73 m^2^	3.95 ± 3.15	4.51 ± 3.12	4.44 ± 3.66	4.48 ± 2.03	4.29 ± 2.75	3.70 ± 3.23	**0.043**
TSAT, %	31.72 ± 16.35	29.14 ± 8.57	12.81 ± 3.79	27.08 ± 6.82	15.62 ± 3.40	38.76 ± 16.79	**<0.001**
SF, ng/mL	1054.11 ± 1061.75	316.84 ± 106.50	64.48 ± 23.56	68.33 ± 18.11	646.85 ± 615.49	1505.40 ± 1150.96	**<0.001**
Hb, g/dL	9.24 ± 1.40	9.67 ± 1.31	9.36 ± 1.45	9.68 ± 1.51	9.19 ± 1.35	9.10 ± 1.41	**<0.001**
MCV, fL	87.11 ± 7.18	87.52 ± 6.79	85.62 ± 7.94	88.51 ± 4.92	86.00 ± 7.21	87.36 ± 7.28	**0.011**
D/P4	0.665 ± 0.139	0.648 ± 0.130	0.662 ± 0.127	0.641 ± 0.160	0.651 ± 0.137	0.673 ± 0.141	**0.113**
MTAC, mL/min/1.73 m^2^	10.40 ± 5.61	9.65 ± 4.53	10.40 ± 4.66	9.56 ± 5.00	9.65 ± 4.82	10.80 ± 6.04	**0.109**
CRP*, mg/L	3.11 ± 4.88	1.77 ± 2.81	2.80 ± 4.40	2.80 ± 4.92	3.99 ± 5.44	3.17 ± 5.03	**0.001**
Adjusted Calcium*, mmol/L	2.22 ± 0.21	2.22 ± 0.20	2.26 ± 0.22	2.20 ± 0.18	2.22 ± 0.19	2.21 ± 0.22	0.525
Phosphate*, mmol/L	1.87 ± 0.54	1.84 ± 0.50	1.95 ± 0.47	1.77 ± 0.40	1.89 ± 0.56	1.87 ± 0.56	0.348
Albumin, g/L	34.81 ± 6.08	36.35 ± 5.35	36.71 ± 5.95	37.40 ± 4.91	34.41 ± 6.00	34.28 ± 6.27	**<0.001**
Vitamin B12*, pg/mL	220.78 ± 237.85	186.06 ± 200.23	137.46 ± 86.95	164.82 ± 69.47	214.04 ± 248.30	234.53 ± 245.97	**0.001**
Folate*, ng/mL	24.46 ± 15.28	24.19 ± 14.46	21.91 ± 9.18	32.30 ± 19.46	23.66 ± 14.04	24.78 ± 15.94	0.569
FG*, mmol/L	6.08 ± 2.31	6.43 ± 3.03	5.55 ± 1.18	6.00 ± 2.84	6.36 ± 2.42	5.89 ± 1.97	**0.001**
HbA1c*, %	6.37 ± 1.24	6.63 ± 1.48	6.02 ± 0.83	5.96 ± 1.05	6.59 ± 1.27	6.22 ± 1.11	**<0.001**
LDLc*, mmol/L	2.34 ± 1.10	2.34 ± 1.13	2.35 ± 1.59	2.11 ± 1.03	2.29 ± 1.03	2.36 ± 1.10	0.794
TG*, mmol/L	1.54 ± 1.13	1.57 ± 1.17	1.57 ± 1.05	1.12 ± 0.40	1.56 ± 1.11	1.53 ± 1.14	0.679

rGFR: residual glomerular filtration rate; Hb: hemoglobin; MCV: mean corpuscular volume; CRP: C-reactive protein; D/P4: dialysate-to-plasma creatinine ratio at 4 h; FG: fasting glucose; LDL-c: low density lipoprotein cholesterol; MTAC, mass transfer area coefficient of creatinine; TG: triglycerides

*Within 90 d before/after start of dialysis.

^a^See text for the definition of each iron status group.

The bold values indicates statistical significance.

### Relation with patient survival

**Figure 1.**
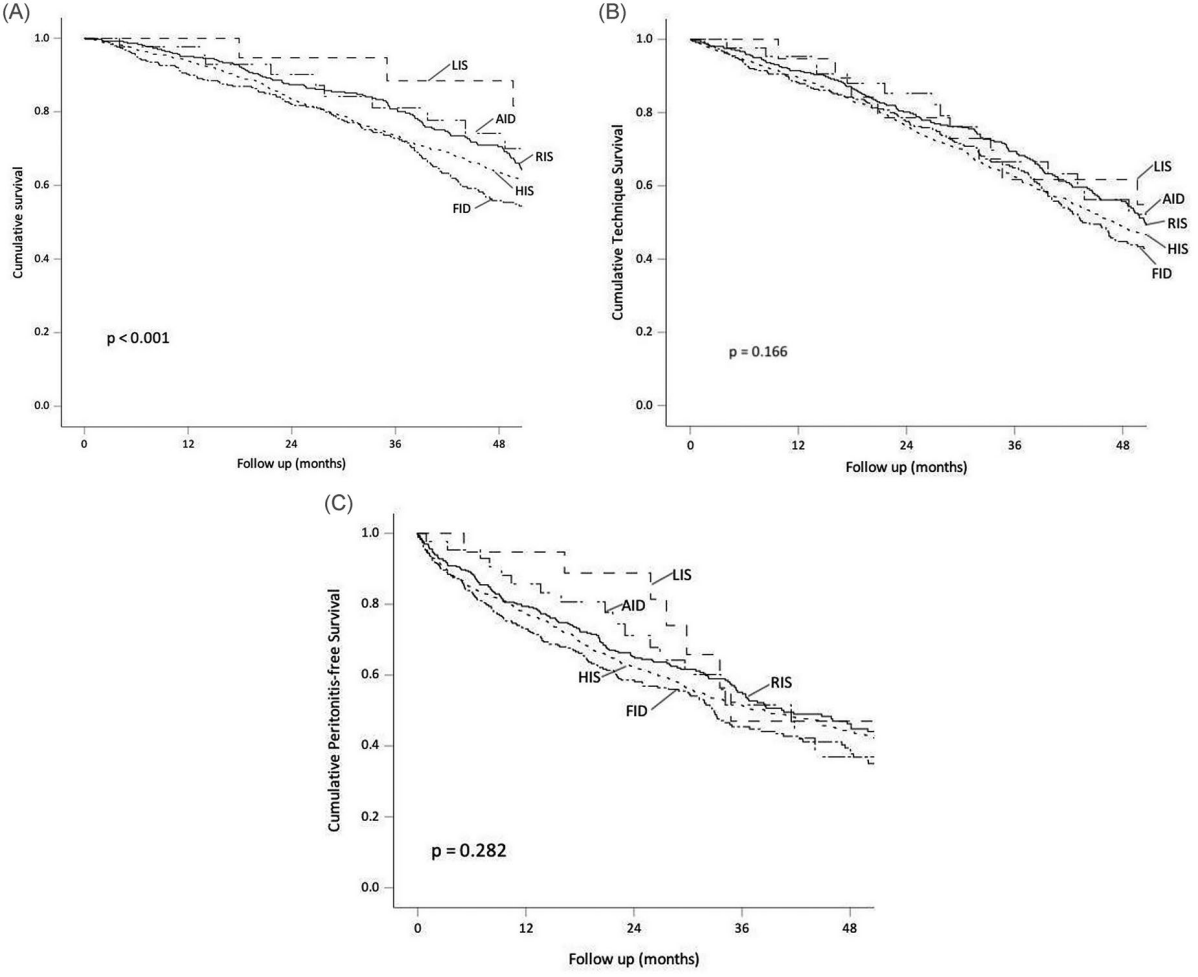
Kaplan–Meier Plots on the relation between baseline iron status and (A) patient survival; (B) technique survival; and (C) peritonitis-free survival. Data were compared by the log-rank test. (RIS: reference iron status; AID: absolute iron deficiency; LIS: low iron storage; FID: functional iron deficiency; HIS: high iron status.).

During a median follow-up of 35.2 (IQR 20.2–55.7) months, 951 (46.9%) patients died. The causes of death were infections other than peritonitis (326 cases), ischemic heart disease (178 cases), sudden cardiac death (103 cases), peritonitis (115 cases), stroke (75 cases), malignancy (29 cases), termination of dialysis (31 cases), other causes (49 cases), undetermined causes (45 case). The Kaplan–Meier plot on patient survival, grouped by the baseline iron status, are shown in Figure 1(A). The 2-year patient survival rates for the RIS, AID, LIS, FID, and HIS groups were 87.4%, 90.2%, 94.7%, 82.0%, and 83.5%, respectively (log-rank test, *p* < 0.001). However, in the multivariate Cox proportional hazard regression analysis, the risk of all-cause mortality was not significantly different between baseline iron status groups after adjusting for other clinical confounders (Table 3). Instead, the risk of mortality was significantly associated with HbA1c (aHR 1.27, 95% CI 1.10–1.46, *p* < 0.001) and oral ferrous use (aHR 1.46, 95% CI 1.01–2.10, *p* = 0.045). Subgroup analysis of the FID group did not show any association between serum CRP level and patient survival (details not shown).

**Table 3.** Cox regression model for the effect of baseline iron status and patient survival.

	Univariate analysis		Multivariable analysis	
Survival	HR (95% C.I.)	*p* Value	HR (95% C.I.)	*p* Value
Iron status at start of PD		**<0.001**		
AID	0.69 (0.38, 1.28)	0.245	0.00 (1.19E + 182)	0.961
LIS	0.71 (0.33, 1.53)	0.384	2.12 (0.27, 16.94)	0.478
FID	1.58 (1.26, 1.98)	**<0.001**	1.01 (0.52, 1.97)	0.967
HIS	1.30 (1.08, 1.57)	**0.007**	1.18 (0.68, 2.07)	0.556
Age (years)	1.02 (1.02, 1.03)	**<0.001**	1.01 (0.99, 1.03)	0.206
Sex (female)	0.74 (0.65, 0.85)	**<0.001**	1.02 (0.70, 1.48)	0.917
Kt/V	0.84 (0.72, 0.98)	**0.022**	0.91 (0.60, 1.19)	0.487
GFR, mL/min/1.73 m^2^	0.98 (0.95, 1.01)	0.152		
CCI	1.17 (1.14, 1.20)	**<0.001**	1.09 (0.99, 1.19)	0.070
Hb, g/dL	0.97 (0.92, 1.01)	0.121		
MCV, fL	1.00 (0.99, 1.01)	0.655		
Thal trait	0.83 (0.51, 1.33)	0.431		
Adjusted Calcium*, mmol/L	0.76 (0.56, 1.03)	**0.076**	0.53 (0.27, 1.07)	0.078
Phosphate*, mmol/L	0.93 (0.83, 1.05)	0.218		
D/P4	1.32 (0.64, 2.72)	0.455		
MTAC, mL/min/1.73 m^2^	1.01 (0.99, 1.02)	0.522		
CRP*, mg/L	1.02 (1.01, 1.04)	**0.002**	1.02 (0.99, 1.06)	0.227
Albumin, g/L	0.94 (0.93, 0.95)	**<0.001**	1.00 (0.97, 1.03)	0.874
Vitamin B12*, pg/mL	1.00 (1.00, 1.00)	0.465		
Folate*, ng/ml	0.99 (0.99, 1.00)	**0.090**	0.99 (0.97, 1.00)	0.146
HbA1c*, %	1.22 (1.15, 1.29)	**<0.001**	1.27 (1.10, 1.46)	**0.001**
LDLc*, mmol/L	0.97 (0.91, 1.04)	0.385		
TG*, mmol/L	1.03 (0.98, 1.09)	0.253		
IV iron use*	1.95 (1.38, 2.75)	**<0.001**	1.41 (0.74, 2.69)	0.303
Oral iron use*	1.12 (0.98, 1.28)	**0.099**	1.46 (1.01, 2.10)	**0.045**
EPO use*	0.91 (0.80, 1.04)	0.180		
Transfused^#^	1.38 (1.22, 1.57)	**<0.001**	0.85 (0.59, 1.22)	0.375

HR: hazard ratio; C.I.: confidence interval; AID: absolute iron deficiency; LIS: low iron storage; FID: functional iron deficiency; HIS: high iron status; GFR: glomerular filtration rate; CCI: Charlson Comorbidity Index; D/P4: dialysate-to-plasma creatinine ratio at 4 h; Hb: hemoglobin; MCV: mean corpuscular volume; CRP: C-reactive protein; LDL-c: low density lipoprotein cholesterol; MTAC: mass transfer area coefficient of creatinine; TG: triglycerides; IV iron: intravenous iron; EPO: erythropoietin

*6 months around the start of dialysis. ^#^Within 90 d before start of dialysis.

The bold values indicates statistical significance.

### Relation with technique survival

During the follow up period, 222 patients were converted to hemodialysis, 102 patients had kidney transplantation, 6 patients had recovery of renal function, 36 patients had transferred to other centers, and 5 patients had lost to follow up. The Kaplan–Meier plot of technique survival, grouped by the baseline iron status, are shown in Figure 1(B). The 2-year technique survival rates for the RIS, AID, LIS, FID, and HIS groups according to the baseline status were 80.5%, 85.2%, 78.6%, 77.6%, and 76.3% respectively (*p* = 0.166). The result of the multivariate Cox proportional hazard regression analysis for the technique survival is summarized in Supplementary Table 1.

### Relation with peritonitis

During the study period, there were a total of 2,099 episodes of peritonitis. The overall peritonitis rate was 0.43 ± 0.97 episode per year; the average time to first peritonitis was 27.15 ± 23.55 months. The Kaplan − Meier plot of peritonitis-free survival, grouped by the baseline iron status, is shown in Figure 1(C). In essence, the 2-year peritonitis-free survival rates for RIS, AID, LIS, FID, and HIS according to the baseline status were 65.2%, 71.2%, 88.8%, 58.6%, and 62.1%, respectively (*p* = 0.282). The result of the multivariate Cox regression analysis for the peritonitis-free survival is summarized in Supplementary Table 2.

### Relation with hospitalization

**Figure 2.**
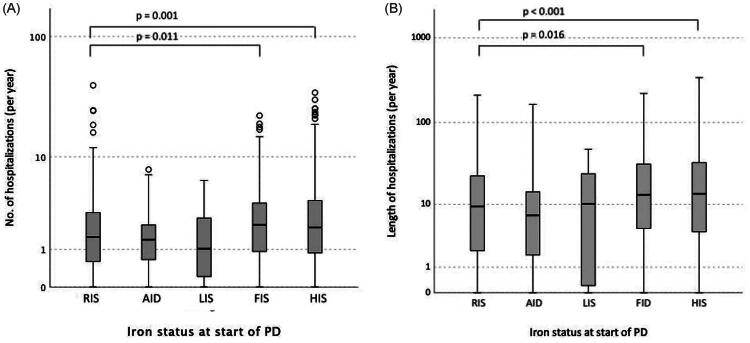
The relation between baseline iron status and (A) number of hospital admission; and (B) duration of hospital stay. Whisker-box plots, with boxes indicating median, 25th and 75th percentiles, whiskers indicating 5th and 95th percentiles. Overall data comparison by Kruskal–Wallis test, with *p* < 0.001 for both. *p* values of *post hoc* analysis, adjusted for multiple comparisons, are depicted on the figure. (RIS: reference iron status; AID: absolute iron deficiency; LIS: low iron storage; FID: functional iron deficiency; HIS: high iron status.).

The average number of hospitalizations was 2.88 ± 3.45 episodes per year of follow-up for the entire cohort, and the average length of hospitalization was 24.99 ± 37.12 d per year. The number of hospitalization and the duration of hospitalization were both significantly different between baseline iron status groups (Kruskal–Wallis test, *p* < 0.001 for both). Post-hoc analysis showed that the FID group was associated with more hospitalization than the other groups (Figure 2). However, with multiple linear regression models, the baseline iron status did not have any independent association with the number of hospital admission or duration of hospitalization after adjusting for other clinical confounding factors (Table 4).

**Table 4.** Multiple linear regression model for the effect of baseline iron status and hospitalizations.

	No. of hospital admission				Duration of hospitalization			
	Univariate model		Multivariate model		Univariate model		Multivariate model	
	Beta (95% C.I.)	*p* Value	Beta (95% C.I.)	*p* Value	Beta (95% C.I.)	*p* Value	Beta (95% C.I.)	*p* Value
Iron status at start of PD								
AID	−0.06 (−0.15, 0.04)	0.234			−0.08 (−0.27, 0.11)	0.394		
LIS	−0.08 (−0.22, 0.06)	0.248			−0.10 (−0.37, 0.18)	0.490		
FID	0.07 (0.03, 0.11)	**0.002**	0.02 (−0.09, 0.12)	0.775	0.14 (0.06, 0.23)	**0.001**	0.04 (−0.17, 0.24)	0.738
HIS	0.07 (0.04, 0.11)	**<0.001**	0.02 (−0.06, 1.11)	0.620	0.16 (0.09, 0.23)	**<0.001**	0.02 (−0.15, 0.20)	0.780
Age (years)	0.00 (0.00, 0.00)	**<0.001**	−0.00 (−0.01, 0.00)	0.380	0.01 (0.01, 0.01)	**<0.001**	−0.00 (−0.01, 0.00)	0.383
Sex (female)	−0.03 (−0.06, −0.01)	**0.019**	−0.04 (−0.11, 0.03)	0.245	−0.06 (−0.12, −0.01)	**0.022**	−0.15 (−0.30, −0.01)	**0.035**
Kt/V	−0.05 (−0.07, −0.02)	**<0.001**	0.01 (−0.03, 0.07)	0.607	−0.12 (−0.17, −0.06)	**<0.001**	−0.00 (−0.11, 0.11)	0.973
GFR, mL/min/1.73 m^2^	−0.01 (−0.02, −0.01)	**<0.001**	−0.02 (−0.04, −0.01)	**0.003**	−0.02 (−0.03, −0.01)	**<0.001**	−0.04 (−0.07, −0.01)	**0.004**
CCI	0.03 (0.02, 0.03)	**<0.001**	0.02 (0.01, 0.04)	**0.005**	0.07 (0.05, 0.08)	**<0.001**	0.05 (0.02, 0.09)	**0.001**
Hb, g/dL	−0.02 (−0.03, −0.01)	**<0.001**	−0.02 (−0.04, 0.00)	0.058	−0.05 (−0.06, −0.03)	**<0.001**	−0.05 (−0.09, −0.01)	**0.021**
MCV, fL	0.00 (−0.002, 0.002)	0.911			0.00 (−0.00, 0.00)	0.748		
Thal trait	−0.03 (−0.12, 0.05)	0.438			−0.10 (−0.27, 0.07)	0.265		
Adjusted Ca*, mmol/L	−0.07 (−0.13, −0.01)	**0.029**	−0.09 (−0.23, 0.04)	0.179	−0.13 (-.0.25, −0.01)	**0.040**	−0.14 (−0.42, 0.13)	0.306
Phosphate*, mmol/L	0.01 (−0.01, 0.03)	0.379			0.01 (−0.04, 0.06)	0.705		
D/P4	1.41 (0.92, 2.15)	0.115			2.19 (0.84, 5.74)	0.110		
MTAC, mL/min/1.73 m^2^	1.01 (1.00, 1.02)	0.124			1.02 (1.00, 1.04)	0.118		
CRP*, mg/L	0.01 (0.00, 0.01)	**0.002**	0.01 (−0.00, 0.01)	0.178	0.01 (0.01, 0.02)	**0.001**	0.02 (0.00, 0.04)	**0.020**
Albumin, g/L	−0.01 (−0.01, −0.01)	**<0.001**	0.00 (−0.00, 0.01)	0.627	−0.02 (−0.03, −0.02)	**<0.001**	0.01 (−0.01, 0.02)	0.329
B12*, pg/mL	0.00 (0.00, 0.00)	**0.002**	3.06E-5 (0.00, 0.00)	0.648	0.00 (0.00, 0.00)	**0.005**	3.73E-6 (0.00, 0.00)	0.978
Folate*, ng/mL	0.00 (−0.00, 0.00)	0.858			−0.00 (−0.00, 0.00)	0.556		
HbA1c*, %	0.04 (0.03, 0.05)	**<0.001**	0.05 (0.02, 0.08)	**<0.001**	0.09 (0.06, 0.11)	**<0.001**	0.10 (0.04, 0.16)	**<0.001**
LDLc*, mmol/L	0.01 (−0.00, 0.02)	0.089			0.02 (−0.01, 0.05)	0.129		
TG*, mmol/L	0.02 (0.01, 0.03)	**0.004**	0.02 (−0.01, 0.05)	0.134	0.03 (0.01, 0.05)	**0.020**	0.05 (−0.00, 0.11)	0.053
IV iron use*	−0.01 (−0.07, 0.05)	0.800			−0.04 (−0.16, 0.08)	0.564		
Oral iron use*	0.01 (−0.02, 0.04)	0.445			0.03 (−0.03, 0.08)	0.318		
EPO use*	−0.05 (−0.08, −0.02)	**<0.001**	−0.05 (−0.12, 0.02)	0.143	−0.13 (−0.18, −0.08)	**<0.001**	−0.09 (−0.23, 0.04)	0.178
Transfused^#^	0.12 (0.10, 0.15)	**<0.001**	0.08 (0.02, 0.14)	**0.011**	0.24 (0.19, 0.29)	**<0.001**	0.17 (0.04, 0.29)	**0.009**

C.I.: confidence interval; AID: absolute iron deficiency; LIS: low iron storage; FID: functional iron deficiency; HIS: high iron status; GFR: glomerular filtration rate; CCI: Charlson Comorbidity Index; D/P4: dialysate-to-plasma creatinine ratio at 4 h; Hb: hemoglobin; MCV: mean corpuscular volume; CRP: C-reactive protein; LDL-c: low density lipoprotein cholesterol; MTAC: mass transfer area coefficient of creatinine; TG: triglycerides; IV iron: intravenous iron; EPO: erythropoietin;

*6 months around the start of dialysis. ^#^Within 90 d before start of dialysis.

The bold values indicates statistical significance.

## Discussion

In this study, we found that HIS and FID were present in 57.5% and 18.8% of incident PD patients, respectively, while AID was found in only 2.4% of the patients. FID at the start of PD was associated with a trend of lower patient survival and higher hospitalization rate, although the associations became insignificant after adjusting for other clinical confounders.

The prevalence of abnormal iron status that we observed was substantially different from previous reports. For example, early studies showed that FID was present in 69% of PD patients [9], while hepatic iron is normal in most PD patients, and iron overload was uncommon [10]. Hepatic magnetic resonance imaging studies revealed that 30.2% patients had several hepatic iron overload [11]. A recent study in Chinese PD patients reported that FID was present in 29.3%, HIS was present in 4.1%, while AID was found in 29.3% [5]. In hemodialysis patients, Plastina et al. [12] reported that FID was present in 36.9%, while Kang et al. [7] noted that FID and HIS were present in 4.1% and 3.9% of their patients, respectively. The reason of the highly variable iron status between patient populations is not entirely clear, and may possibly be related to the differences in dietary pattern and coexisting chronic inflammatory conditions.

There are good theoretical reasons to expect body iron status to be related to the clinical outcome of PD patients. Both iron overload and iron deficiency could lead to and worsen heart failure [13]. Iron overload causes damage in cardiomyocytes [14] and liver parenchyma [15]. On the other hand, FID is a characteristic feature of chronic systemic inflammatory state [16], which is a well-known risk factor of cardiovascular disease [17]. Although serum CRP level was measured and did not appear as an independent predictor of survival (Table 3), we did not measure other inflammatory markers to validate the (lack of) association. Nonetheless, our previous study on a separate cohort of PD patients did not find any association between serum CRP and patient survival [18]. Because of the limitations in the original study design, we did not have any assessment of endothelial function (e.g., brachial artery flow-mediated dilation), which would give more details to the cardiovascular risk of the patients.

There is a wealth of literature that explored the association between iron status and the clinical outcome of dialysis patients, but they were mostly focused on hemodialysis. From a study in Korea, patients with normal iron status were found to have the most favorable survival rates while patients with FID or high iron storage had lower patient survival rates [7]. A study from Japan reported a U-shaped association between ferritin levels and the risk of all-cause mortality [19]. Another multi-national study also reported that high ferritin levels were associated with higher mortality [20]. Similar findings were also noted in pre-dialysis CKD patients; Fujisawa et al. [21] reported that lower and higher ferritin levels were both independent risk factors for CKD progression.

Unlike hemodialysis, there were few studies that examined the relation between iron status and the clinical outcome of PD patients. Kuo et al. [22] reported that serum ferritin level above 800 ng/mL was associated with a higher all-cause mortality, and an iron saturation between 20 and 50% was associated with the lowest all-cause mortality. Similarly, Luo et al. [5] found that both FID and HIS were independent risk factors for all-cause mortality, while only HIS was associated with an increased risk of cardiovascular mortality. The result of this study is actually similar to those reported by other groups [5,22], although we found that the prognostic effect of the baseline iron status became insignificant after multivariable analysis, probably because we had more extensive adjustment for clinical confounders in our computation. Notably, since diabetes and its complications are known predictors of poor outcomes in PD patients, the higher prevalence of diabetes in the FID group may have influenced the observed associations and limited the ability to isolate the impact of iron status on patient outcomes.

In this study, we did not observe any correlation between body iron status and the risk of peritonitis. In contrast, *in vitro* data suggested that transferrin within peritoneal dialysate may be an indicator of the potential for bacterial growth, and represent a possible risk factor of PD-related peritonitis [23]. The previous study of Diao et al. [6] further showed that patients with AID was associated with both the risk and treatment failure of peritonitis, while patients with HIS was associated with treatment failure of peritonitis. However, the overall peritonitis rate in that report was around 0.12 episode per patient-year of treatment [6], which is drastically lower than our unit as well as most of the other centers in the world [24], suggesting that their study population was highly selected and their result may not be applicable to the others.

There were several limitations of this study. First, there is an inherent limitation associated with the analysis of iron stores restricted at the initiation of dialysis. During the early years of dialysis, a considerable proportion of patients may have received iron supplement, which could have influenced survival outcomes and potentially resulted in the reclassification of initial iron stores categories. Further studies are necessary to evaluate the role of iron treatment received during the early years of dialysis on the clinical outcome, as well as its impact on the classification of iron stores. Second, our study was an observation study, causal relation between iron status and clinical outcomes could not be testified. In addition, clinical markers of body iron status are far from perfect. Specifically, although serum ferritin concentrations may adequately reflect bone marrow iron stores [25], it does not reflect iron overload in liver parenchyma or myocardium. Moreover, serum ferritin is known to be a positive acute-phase protein whereas transferrin is a negative acute-phase protein [26]. Unfortunately, we were not able to describe the dosage of ESA because of the variable preparation and frequent change in dosage. We did not have a comprehensive assessment of systemic inflammation or measurement of circulating hepcidin level. Similarly, during the initial two or three years of dialysis, patients could have received substantial iron supplements, including oral and/or IV doses, which may have exerted a considerable influence on the clinical outcomes and potentially leading to the reclassification of initial iron stores categories in a significant proportion of patients. In this study, we took into consideration the effect of iron supplement 6 months around the initiation of dialysis. Further studies are required to explore the effect of iron supplement as a time-dependent covariate on the clinical outcome of PD patients. Nonetheless, our study provided important data on the epidemiology of body iron status in a cohort of unselected PD population.

In summary, HIS and FID are both common in incident PD patients. FID at the start of PD was associated with a trend of lower patient survival and higher hospitalization rate, although the associations became insignificant after adjusting for other clinical confounders. Further studies are required to determine the threshold iron saturation or ferritin level that would guide iron supplement therapy.

## Supplementary Material

Supplemental Material
