# Supplementary material for: The association of iron status on clinical outcomes in peritoneal dialysis: a retrospective study over 10 years
Source: Ren Fail. 2025 Oct 9;47(1):2567522. doi: 10.1080/0886022X.2025.2567522 (PMC12512763; doi:10.1080/0886022X.2025.2567522)
Supplement: Supplemental Material [file IRNF_A_2567522_SM8870.docx]

# Supplementary Table 1. Cox regression model for the effect of baseline iron status and technique survival

| Technique survival | Univariate analysis | | Multivariate analysis | |
| --- | --- | --- | --- | --- |
|  | HR (95% C.I.) | P value | HR (95% C.I.) | P value |
| Iron status at start of PD |  | 0.168 |  |  |
| AID | 0.91 (0.58, 1.44) | 0.690 |  |  |
| LIS | 0.88 (0.49, 1.57) | 0.661 |  |  |
| FID | 1.24 (1.02, 1.50) | **0.033** |  |  |
| HIS | 1.15 (0.98, 1.35) | **0.089** |  |  |
| Age (years) | 0.10 (0.99, 1.00) | **0.093** | 0.99 (0.97, 1.01) | 0.200 |
| Gender (Female) | 0.71 (0.63, 0.79) | **<0.001** | 0.87 (0.63, 1.20) | 0.395 |
| Kt/V | 0.74 (0.64, 0.85) | **<0.001** | 0.85 (0.68, 1.07) | 0.172 |
| GFR, ml/min/1.73 m^2^ | 0.99 (0.96, 1.01) | 0.299 |  |  |
| CCI | 1.07 (1.05, 1.10) | **<0.001** | 1.06 (0.98, 1.15) | 0.141 |
| Hb, g/dl | 0.97 (0.94, 1.01) | 0.170 |  |  |
| MCV, fL | 1.00 (0.99, 1.01) | 0.615 |  |  |
| Thal trait | 0.79 (0.52, 1.19) | 0.253 |  |  |
| Adjusted Calcium*, mmol/L | 0.74 (0.57, 0.97) | **0.030** | 0.79 (0.42, 1.48) | 0.463 |
| Phosphate*, mmol/L | 1.13 (1.03, 1.25) | **0.013** | 1.01 (0.76, 1.34) | 0.961 |
| D/P4 | 1.10 (0.59, 2.06) | 0.768 |  |  |
| MTAC, ml/min/1.73m^2^ | 1.00 (0.98, 1.02) | 0.935 |  |  |
| CRP*, mg/L | 1.01 (1.00, 1.03) | **0.089** | 1.01 (0.99, 1.04) | 0.385 |
| Albumin, g/L | 0.96 (0.95, 0.97) | **<0.001** | 1.01 (0.99, 1.01) | 0.422 |
| Vitamin B12*, pg/ml | 1.00 (1.00, 1.00) | 0.690 |  |  |
| Folate*, ng/ml | 0.99 (0.99, 1.00) | **0.056** | 1.00 (0.99, 1.01) | 0.823 |
| HbA1c*, % | 1.09 (1.04, 1.15) | **<0.001** | 1.10 (0.97, 1.25) | 0.144 |
| LDLc*, mmol/L | 1.02 (0.96, 1.07) | 0.589 |  |  |
| TG*, mmol/L | 1.04 (0.99, 1.09) | **0.091** | 1.06 (0.95, 1.18) | 0.332 |
| IV iron use* | 2.14 (1.61, 2.86) | **<0.001** | 1.91 (1.15, 3.18) | **0.013** |
| Oral iron use* | 1.04 (0.92, 1.16) | 0.540 |  |  |
| EPO use* | 1.02 (0.91, 1.14) | 0.712 |  |  |
| Transfused^#^ | 1.17 (1.05, 1.31) | **0.005** | 1.00 (0.75, 1.34) | 0.997 |

HR: hazard ratio; C.I.: confidence interval; AID: Absolute iron deficiency; LIS: Low iron storage; FID: Functional iron deficiency; HIS: High iron status; GFR: glomerular filtration rate; CCI: Charlson Comorbidity Index; Hb: hemoglobin; MCV: mean corpuscular volume; CRP: C-reactive protein; dialysate-to-plasma creatinine ratio at 4 hour; LDL-c: low density lipoprotein cholesterol; MTAC, mass transfer area coefficient of creatinine; TG: triglycerides; IV iron: intravenous iron; EPO: erythropoietin; *6 months around the start of dialysis. ^#^ Within 90 days before start of dialysis. ^a^p <0.05 is considered statistically significant.

# Supplementary Table 2. Cox regression model for the effect of baseline iron status and peritonitis-free survival

| Peritonitis-free survival | Univariate analysis | | Multivariate analysis | |
| --- | --- | --- | --- | --- |
|  | HR (95% C.I.) | P value | HR (95% C.I.) | P value |
| Iron status at start of PD |  | 0.287 |  |  |
| AID | 1.07 (0.69, 1.66) | 0.776 |  |  |
| LIS | 0.66 (0.31, 1.41) | 0.284 |  |  |
| FID | 1.31 (.098, 1.49) | **0.074** |  |  |
| HIS | 1.07 (0.91, 1.27) | 0.417 |  |  |
| Age (years) | 1.01 (1.00, 1.02) | **<0.001** | 1.00 (0.99, 1.01) | 0.828 |
| Gender (Female) | 0.92 (0.81, 1.04) | 0.191 |  |  |
| Kt/V | 0.97 (0.84, 1.11) | 0.633 |  |  |
| GFR, ml/min/1.73 m^2^ | 0.99 (0.96, 1.02) | 0.326 |  |  |
| CCI | 1.07 (1.04, 1.10) | **<0.001** | 1.01 (0.96, 1.08) | 0.650 |
| Hb, g/dl | 0.96 (0.92, 1.01) | **0.085** | 0.97 (0.90, 1.05) | 0.423 |
| MCV, fL | 1.00 (0.99, 1.01) | 0.550 |  |  |
| Thal trait | 0.76 (0.48, 1.19) | 0.233 |  |  |
| Adjusted Calcium*, mmol/L | 0.94 (0.69, 1.28) | 0.709 |  |  |
| Phosphate*, mmol/L | 1.03 (0.92, 1.15) | 0.591 |  |  |
| D/P4 | 1.64 (0.84, 3.19) | 0.146 |  |  |
| MTAC, ml/min/1.73m^2^ | 1.01 (0.99, 1.03) | 0.265 |  |  |
| CRP*, mg/L | 1.02 (1.00, 1.04) | **0.029** | 1.01 (0.99, 1.03) | 0.215 |
| Albumin, g/L | 0.97 (0.96, 0.98) | **<0.001** | 0.99 ().97, 1.01) | 0.314 |
| Vitamin B12*, pg/ml | 1.00 (1.00, 1.00) | 0.907 |  |  |
| Folate*, ng/ml | 1.00 (0.99, 1.00) | 0.521 |  |  |
| HbA1c*, % | 1.09 (1.02, 1.15) | **0.007** | 1.10 (1.00, 1.19) | **0.041** |
| LDLc*, mmol/L | 0.98 (0.92, 1.05) | 0.583 |  |  |
| TG*, mmol/L | 0.99 (0.93, 1.05) | 0.688 |  |  |
| IV iron use* | 0.95 (0.68, 1.34) | 0.780 |  |  |
| Oral iron use* | 1.02 (0.90, 1.16) | 0.721 |  |  |
| EPO use* | 0.94 (0.83, 1.06) | 0.306 |  |  |
| Transfused^#^ | 1.27 (1.12, 1.43) | **<0.001** | 1.05 ().84, 1.30) | 0.694 |

HR: hazard ratio; C.I.: confidence interval; AID: Absolute iron deficiency; LIS: Low iron storage; FID: Functional iron deficiency; HIS: High iron status; GFR: glomerular filtration rate; CCI: Charlson Comorbidity Index; Hb: hemoglobin; MCV: mean corpuscular volume; CRP: C-reactive protein; dialysate-to-plasma creatinine ratio at 4 hour; LDL-c: low density lipoprotein cholesterol; MTAC, mass transfer area coefficient of creatinine; TG: triglycerides; IV iron: intravenous iron; EPO: erythropoietin; *6 months around the start of dialysis. ^#^ Within 90 days before start of dialysis. ^a^p <0.05 is considered statistically significant.
